# Smoking behaviors before and after implementation of a smoke-free legislation in Guangzhou, China

**DOI:** 10.1186/s12889-015-2353-6

**Published:** 2015-09-29

**Authors:** Xiaohua Ye, Sidong Chen, Zhenjiang Yao, Yanhui Gao, Ya Xu, Shudong Zhou, Zhengwei Zhu, Liang Wang, Yi Yang

**Affiliations:** Department of Epidemiology and Biostatistics, School of Public Health, Guangdong Pharmaceutical University, Guangzhou, China; Guangzhou Association on Tobacco Control, Guangzhou, China; College of Public Health, East Tennessee State University, Johnson, USA

**Keywords:** Smoke-free, Tobacco control, Smoking

## Abstract

**Background:**

According to the partial smoke-free legislation implemented on 1 September 2010 in Guangzhou, China, smoke-free did not cover all indoor areas. Some places have a full smoking ban (100 % smoke-free), other places have a partial smoking ban, and homes have no ban. This study aimed to compare the smoking behaviors before and after implementation of a smoke-free legislation.

**Method:**

A repeated cross-sectional survey was conducted on smoking-related behaviors with a total of 4,900 respondents before, and 5,135 respondents after the legislation was instituted. For each wave of the survey, a three-stage stratified sampling process was used to obtain a representative sample. Pearson’s Chi-square test was used to determine differences of smoking prevalence and quit ratio between the two samples. Logistic regression models were used to examine the associations of a smoke-free legislation with smoking behaviors.

**Results:**

The overall daily smoking rate declined significantly from 20.8 % to 18.2 % (*p* < 0.05), especially among those aged 15–24 years. The quit ratios increased significantly (from 14.5 % to 17.9 %), but remained low among 15–44 year olds. The overall self-reported smoking behaviors in locations with a full smoking ban decreased significantly from 36.4 % to 24.3 % with the greater drops occurring in cultural venues, public transport vehicles, and government offices. Smoking in places with partial smoking bans remained high (89.6 % vs. 90.4 %), although a slight decrease was observed in some of these areas. The implementation of a smoke-free legislation did not lead to more smoking in homes (91.0 % vs 89.4 %), but smoking in homes remained high.

**Conclusions:**

These findings highlight the urgent need for a comprehensive smoke-free legislation covering all public places in Guangzhou, simultaneously educational interventions and campaigns promoting voluntary changes in home smoking need to occur.

## Background

Tobacco use substantially increases the risk of dying from cancers, heart diseases, stroke and chronic respiratory diseases, and has been the second leading risk factor for deaths worldwide [1]. It is noteworthy that tobacco use is increasing in many low- and middle-income countries [2]. By 2030, if current patterns of use persist, tobacco will kill more than 8 million people worldwide each year, and 80 % of these premature deaths will occur in low- and middle-income countries [2]. One such country, the world’s largest producer, consumer and victim of tobacco, is China. A recent study indicated that China was home to 301 million smokers (45.5 % of the world’s smokers), only 16 % of current smokers were looking to quit in the coming year [3]. Approximately one million people die every year due to direct or indirect tobacco-related deaths [4].

The findings from studies conducted in several countries indicate that smoke-free legislations can improve indoor air quality, reduce tobacco use and decrease hospital admissions attributed to acute coronary syndrome [5–8]. Although so far no national smoke-free law exists in China, Guangzhou was one of the earliest cities to implement a partial smoke-free legislation, beginning September 1, 2010. According to the legislation, smoke-free did not cover all indoor areas. Some places (including cultural venues, public transportation vehicles, government offices, commercial venues, medical facilities, schools, and stadiums) have a full smoking ban (100 % smoke-free, without designated smoking rooms), other places (including workplaces, restaurants, hotels, cafes, bars, nightclubs, amusement parks, and waiting rooms of transportation vehicles) have a partial smoking ban (with designated smoking rooms), and homes have no smoking ban. Although Guangzhou did not adopt a 100 % smoke-free policy, the partial smoke-free legislation was the most strict tobacco control policy in China at that time.

Even though a smoke-free legislation can be a powerful public health intervention, little is known about the impacts of a smoke-free legislation on smoking behaviors in full, partial and no smoking ban places in Guangzhou, China. Additionally, before the legislation was implemented, there was concern that people might transfer their smoking from public places to their homes. Therefore, the present study aimed to address the following three questions: (1) Were there differential associations of a smoke-free legislation with smoking behaviors in full and partial smoking ban places in Guangzhou, China? (2) Would smokers transfer their smoking behaviors from public places and workplaces to their homes? (3) Did indicators of smoking prevalence decrease and quit ratios increase in Guangzhou after the legislation was implemented?

## Methods

### Sampling design

Two epidemiological, observational and cross-sectional surveys were conducted in Guangzhou, China. The methods of the survey are described in detail elsewhere [9]. Briefly, a three-stage stratified sampling process was employed to obtain an independent, representative sample. The field work for the baseline survey was undertaken in May 2009, before the implementation of the smoke-free legislation. The evaluation survey was conducted in May 2011, 9 months after implementation of the smoke-free legislation. A total of 4,930 participants were interviewed in the baseline survey, and 5,156 participants were interviewed in the evaluation survey.

### Study variables

The primary outcome variables were smoking prevalence, quit ratio, and smoking behaviors in different kinds of venues. Accordingly, the survey instrument contained three major sections: 1) smoking prevalence, 2) quit ratio and 3) smoking behaviors. To determine the prevalence of smoking, individuals were asked if they were current smokers (a person who has smoked daily or occasionally in the last 30 days for at least 6 months) or former smokers (a person who has a history of smoking for at least 6 months and currently has stopped). To assess smoking behaviors, current smokers were asked a series of questions to determine their travel history (i.e. cultural venues, public transportation vehicles, government offices, commercial venues, medical facilities, schools, stadiums, workplaces, restaurants, hotels, cafes, bars, nightclubs, amusement parks, waiting rooms of transportation vehicles and homes) for the past two weeks and if smoking had occurred at that particular location. To evaluate quitting behaviors, the quit ratio was estimated by taking the ratio of the number of former smokers to the number of ever smokers [10]. The main predictor variable was the implementation of the smoke-free legislation.

### Data collection and quality control

Interviewers were enrolled voluntarily from third- and fourth-year undergraduate students in the School of Public Health of Guangdong Pharmaceutical University, China. All interviewers were trained to ensure that the operation procedures were identical across all areas. After obtaining informed consent verbally, eligible respondents were asked to complete a face-to-face survey by the trained interviewers.

### Data analysis

All data were entered in duplicate into the EpiData version 3.1 database, and data entry screens were used to revise incorrect entries. Descriptive statistics were conducted for the two samples. The data from smoking prevalence and quit ratios were examined by sex and age, and differences between the two samples were determined using the Pearson’s chi-square test. Separate logistic regression models were used to examine the associations of a smoke-free legislation with smoking behaviors. The two-sided p-value for statistical significance was defined as *p* < 0.05. To account for sampling design and weight in the estimation procedures, statistical analyses were conducted with weighted data, except for those otherwise specified. All statistical analyses were conducted using STATA version 13.0 (StataCorp LP, College Station, Texas, USA).

### Ethical approval

The study was approved by the ethics committee of Guangdong Pharmaceutical University, and this survey was qualified as involving no risks to participants. A verbal informed consent regarding the goals of the study and the willingness to participate in the study was given by the participants.

## Results

### Demographic characteristics

In the baseline survey conducted in 2009 (before implementation of the smoke-free legislation), a total of 5409 participants were interviewed, of whom 4930 (91.1 %) were willing to participate and 4900 (90.6 %) provided complete data. In the evaluation survey conducted in 2011 (after implementation of the smoke-free legislation), a total of 5614 participants were interviewed, of whom 5156(91.8 %) were willing to participate and 5135(91.5 %) provided complete data. The demographic characteristics were similar in both samples (Table 1) and no significant differences in gender (*p* = 0.255) and age (*p* = 0.313) were detected. Notably, both samples represent the adult population in Guangzhou sufficiently, with the exception of a significant oversampling of females aged 45–54 years in both samples and females aged 55–64 years only in the 2011 sample (Table 2). This discrepancy was adjusted by applying the weighing techniques.

**Table 1:** Demographic characteristics of all participants according to survey waves ((baseline vs evaluation survey), in Guangzhou, China

Variables	Number	Baseline survey		Evaluation survey		*χ* ^2^	*p*
		n_1_	%	n_2_	%		
Gender							
Male	4418	2129	43.5	2289	44.6	1.29	0.255
Female	5617	2771	56.5	2846	55.4		
Age(years)							
15–24	1713	816	16.6	897	17.5	5.93	0.313
25–34	1921	926	18.9	995	19.4		
35–44	2060	1013	20.7	1047	20.4		
45–54	2022	997	20.3	1025	20.0		
55–64	1238	589	12.1	649	12.6		
65+	1081	559	11.4	522	10.1		

All estimates are unweighted

n, number of participants in both surveys; n_1_, number of participants in the baseline survey; n_2_, number of participants surveyed in the evaluation survey; %, the proportion of participants surveyed

**Table 2:** Demographic characteristics of samples according to survey waves (baseline vs evaluation survey) and population according to a census in 2009 in Guangzhou, China

Gender and age	Baseline survey n_1_ (%)	Evaluation survey n_2_ (%)	2009 Population^a^ n (%)
Males			
15–24 years	433 (8.8)	496 (9.7)	722862 (10.6)
25–34 years	409 (8.4)	522 (10.2)	660577 (9.7)
35–44 years	427 (8.7)	496 (9.7)	696426 (10.2)
45–54 years	363 (7.4)	342 (6.7)	601859 (8.8)
55–64 years	229 (4.7)	220 (4.3)	390965 (5.7)
65+ years	272 (5.5)	212 (4.1)	364886 (5.3)
Females			
15–24 years	383 (7.8)	401 (7.8)	667648 (9.8)
25–34 years	517 (10.5)	473 (9.2)	631947 (9.3)
35–44 years	586 (12.0)	551 (10.7)	693369 (10.2)
45–54 years	**634 (12.9)**	**683 (13.3)**	573130 (8.4)
55–64 years	360 (7.4)	**429 (8.3)**	399627 (5.8)
65+ years	287 (5.9)	310 (6.0)	425490 (6.2)

All estimates are the proportion of participants surveyed and unweighted

Significant difference in baseline survey and evaluation survey samples from the 2009 population is highlighted by boldfacing and underlining them (*p* < 0.05 level)

^a^Source from Guangzhou Public Security Bureau

### Smoking prevalence

Smoking prevalence, stratified by sex and age, is reported in Table 3 for both samples. The overall daily smoking rate decreased significantly after implementation of the smoke-free legislation (from 20.8 % in the baseline survey to 18.2 % in the evaluation survey; *p* < 0.01). The reduction in daily smoking rate achieved statistical significance among 15–24 year olds, also within age group, significant reductions in smoking were observed in males (from 27.1 % to 20.4 %; *p* = 0.016) and females (from 2.2 % to 0 %; *p* = 0.003). The change for occasional smokers remained relatively constant and was not statistically significant (1.2 % in baseline survey vs 1.1 % in the evaluation survey; *p* =0.706).

**Table 3:** Smoking prevalence (%) among adults according to survey waves (baseline vs evaluation survey), in Guangzhou, China

Age and gender	Daily smoker		Occasional smoker		Former smoker		Ever smoker		Quit ratio	
	Baseline	Evaluation	Baseline	Evaluation	Baseline	Evaluation	Baseline	Evaluation	Baseline	Evaluation
Total	20.8	**18.2**	1.2	1.1	3.7	4.2	25.6	**23.5**	14.5	**17.9**
15–24 years	15.2	**10.7**	2.0	1.0	0.4	0.9	17.5	**12.6**	2.3	**7.1**
25–34 years	19.3	16.4	1.4	1.1	1.4	2.5	22.1	20.0	6.3	**12.5**
35–44 years	21.0	20.8	0.5	**1.5**	1.8	2.5	23.3	24.9	7.7	10.0
45–54 years	28.4	29.6	1.0	1.0	7.1	**4.9**	36.4	35.5	19.5	**13.8**
55–64 years	27.4	26.2	0.5	0.8	8.6	9.7	36.4	36.7	23.6	26.4
65+ years	17.2	**12.2**	1.2	0.7	16.3	18.6	34.8	31.4	46.8	**59.2**
Males	37.4	34.3	2.0	1.9	6.6	7.5	45.7	43.6	14.4	**17.2**
15–24 years	27.1	**20.4**	3.5	1.9	0.6	1.7	31.2	**24.0**	1.9	**7.1**
25–34 years	35.5	29.8	2.6	1.7	2.7	4.4	40.8	35.9	6.6	**12.3**
35–44 years	37.9	41.4	0.9	**2.7**	3.3	4.9	42.1	**48.9**	7.8	10.0
45–54 years	48.6	52.9	1.7	1.8	12.4	8.0	62.7	62.7	19.8	**12.8**
55–64 years	47.5	49.3	0.8	1.3	14.1	17.0	62.4	67.5	22.6	25.2
65+ years	28.6	23.1	1.5	0.9	29.0	34.2	59.0	58.2	49.2	**58.8**
Females	1.6	**0.5**	0.2	0.3	0.3	**0.7**	2.1	1.5	14.3	**46.7**
15–24 years	2.2	**0.0**	0.4	0.1	0.0	0.0	2.6	**0.1**	0.0	0.0
25–34 years	0.6	0.6	0.0	0.4	0.0	0.3	0.7	1.3	0.0	**23.1**
35–44 years	1.2	0.3	0.0	0.4	0.1	0.1	1.3	0.8	7.7	**12.5**
45–54 years	2.0	0.8	0.0	0.1	0.2	0.9	2.2	1.8	9.1	**50.0**
55–64 years	1.2	0.4	0.0	0.3	1.3	1.6	2.5	2.3	52.0	**69.6**
65+ years	4.2	2.3	1.0	0.6	1.9	4.4	7.1	7.2	26.8	**61.1**

All estimates are weighted

Quit ratio, the ratio of former smokers to ever smokers

Significant difference between baseline and evaluation sample is highlighted by boldfacing and underlining them (*p* < 0.05 level)

### Quit ratio

After implementation of the smoke-free legislation, the quit ratio increased significantly in male smokers (from 14.4 % to 17.2 %; *p* = 0.028; Table 3), and this increase was marked among 15–24, 25–34, and 65+ years old males. Of note, the quit ratio among 15–34 year old males increased because of increasing rates of former smokers and decreasing rates of ever-smokers, and the quit ratio among 65+ year old males increased only because of increasing rates of former smokers. After implementation of the smoke-free legislation, the quit ratio increased more than three-folds in female smokers (from 14.3 % to 46.7 %; *p* < 0.001; Table 3), and this increase was marked among 25+ year old females. The quit ratio among 45–64 year old females increased because of increasing rates of former smokers and decreasing rates of ever-smokers, the quit ratio among 24–34 and 65+ year old females increased only because of increasing rates of former smokers, but the quit ratio among 35–44 year old females increased only because of decreasing rates of ever-smokers. However, the quit ratio remained low among 15–44 year old males and females.

### Smoking behaviors in different kinds of venues

In places where the full smoking ban were implemented, the self-reported overall smoking behaviors decreased significantly (from 36.4 % to 24.3 %; *p* < 0.05; Table 4). The largest impact was observed in both cultural venues (from 22.2 % to 8.7 %; *p* < 0.05) and public transport vehicles (from 10.7 % to 4.2 %; *p* < 0.05) with a 60.8 % reduction. A significant decline also occurred in government offices (from 48.3 % to 24.8 %; *p* < 0.05) with a 48.7 % reduction. Of note, smoking behaviors remained high in any types of partial smoking ban places (Table 4), and a significant decline was observed only in workplaces (from 78.8 % to 64.5 %; *p* < 0.05), restaurants (from 85.3 % to 75.1 %; *p* < 0.05), and hotels (from 83.4 % to 75.6 %; *p* < 0.05). Although the smoke-free regulation did not cover in home environment, the legislation did not lead to more smoking behaviors in homes (91.0 % in baseline survey vs 89.4 % in evaluation survey; *p* = 0.138; Table 4). It was noteworthy that smoking in homes remained high.

**Table 4:** Self-reported smoking of current smokers in the last 2 weeks according to survey waves (baseline vs evaluation survey), in Guangzhou, China

Extent of smoking restriction, venues	Baseline survey		Evaluation survey		Reduction (%)	aOR(95 % CI) for smoking ban	*p*
	n_1_	Smoking (%)	n_2_	Smoking (%)			
Full smoking ban	763	36.4	839	24.3	33.2	0.56(0.38 to 0.82)	0.022
Cultural venues	168	22.2	194	8.7	60.8	0.34(0.17 to 0.68)	0.022
Public transport vehicles	582	10.7	698	4.2	60.8	0.38(0.20 to 0.73)	0.023
Government offices	165	48.3	158	24.8	48.7	0.35(0.13 to 0.93)	0.044
Commercial venues	584	15.8	723	7.6	51.9	0.42(0.16 to 1.07)	0.057
Medical facilities	210	21.5	236	14.0	34.9	0.58(0.26 to 1.31)	0.102
Stadiums	184	42.6	189	29.6	30.5	0.62(0.26 to 1.46)	0.137
Primary/secondary schools	213	21.2	193	19.8	6.6	1.08(0.34 to 3.44)	0.813
Universities	91	31.4	99	29.4	6.4	0.96(0.49 to 1.86)	0.802
Partial smoking ban	842	89.6	873	90.4	−0.9	1.09(0.63 to 1.91)	0.562
Workplaces	510	78.8	581	64.5	18.1	0.46(0.30 to 0.70)	0.015
Restaurants	629	85.3	745	75.1	12.0	0.49(0.26 to 0.91)	0.038
Hotels	188	83.4	230	75.6	9.4	0.67(0.47 to 0.96)	0.041
Cafes/bars/nightclubs	298	90.5	333	89.7	0.9	1.09(0.31 to 3.80)	0.804
Amusement parks	425	66.4	562	63.7	4.1	0.85(0.48 to 1.50)	0.349
Waiting room of transport vehicles	542	48.6	680	49.9	−2.7	1.05(0.43 to 2.54)	0.838
No ban							
Home	882	91.0	877	89.4	1.8	0.78(0.51 to 1.21)	0.138

aOR, adjusted OR; n, number of visitors who visited venues in the last 2 weeks. Smoking(%), weighted ratio of smokers (who smoked in venues) to visitors

Gender and age have been controlled for in the multiple logistic regression models. The survey sample size of current smokers (n) is unweighted while other estimates are weighted

## Discussion

After the implementation of the partial smoke-free legislation began in September 2010 in Guangzhou, China, the self-reported smoking behaviors reduced more significantly in full smoking ban places (from 36.4 % to 24.3 %) than in partial smoking ban places (89.6 % in the baseline survey vs 90.4 % in the evaluation survey), and this legislation did not lead to more smoking in homes (91.0 % vs 89.4 %). The daily smoking prevalence declined significantly (from 20.8 % to 18.2 %), especially among 15–24 year olds, and the quit ratios increased significantly (from 14.5 % to 17.9 %). But smoking in banned places and the home environment still remained high, and the quit ratios remained low.

Studies conducted in several countries have shown that smoke-free legislations can reduce smoking-related behaviors [5–8, 11, 12]. The reduction in smoking occurs, likely because the smoke-free legislation increases support for regulating smoking, reduces the social acceptability of smoking, limits opportunities for smoking, and leads to less socially cued smoking [12–14]. Moreover, there is evidence that the comprehensive smoke-free legislation (i.e., 100 % smoke-free legislation, without designated smoking rooms) has a greater effect on reducing smoking behaviors than the partial smoke-free legislation [15, 16]. This study found a significant reduction in smoking behaviors in full smoking ban places, especially among cultural venues and public transport vehicles. Consistent with previous studies [14–16], it is disappointing that smoking behaviors in these venues were not eliminated, but were still at a high level after implementation of the legislation. It was noteworthy that smoking behaviors in government offices and stadiums started from a high level in the baseline survey (43-48 %) and was still high in the evaluation survey (25-30 %). This observation may be due to poor compliance with the smoke-free legislation in these venues. More disappointing was that smoking behaviors in partial smoking ban places (89.6 % vs 90.4 %) were still remarkably high after the implementation of a partial smoke-free legislation, due to the permissiveness of setting smoking rooms in these places. Notably, very few respondents (1-2 %) reported smoking in workplaces, pubs, cafes or other enclosed public places in England after the implementation of comprehensive smoke-free legislation covering all enclosed public places and workplaces [17].

It is quite disappointing from a public health point of view, given that in both types of venues there were only small decreases of smoking in Guangzhou following the smoke-free legislation. One of the important reasons is that the Guangzhou government did not introduce a comprehensive smoke-free legislation since policymakers regarded the implementation of a partial smoke-free law as more feasible and practical in Guangzhou than a total ban. To note, a full smoking ban implemented in certain venues produced comparatively low smoking rates (10.7 %-48.3 %), while venues with a partial smoking ban revealed high smoking rates (48.6 %-90.5 %), indicating an unwillingness of the policymakers to implement tougher policies. The effectiveness of a smoke-free legislation also required enforcement efforts and compliance from smokers and managers in venues. In Guangzhou, the law enforcement departments, tasked with smoking control, have been ineffective in their efforts. To our surprise, no one (including smokers and managers in the venues) was fined until May 2011, 9 months after the implementation of a smoke-free legislation. This study showed high rates of smoking in public places during the last 2 weeks, indicating low compliance with the smoking regulation. Therefore, increasing the compliance among smokers is the first step with a possible solution to include increasing the fine amount which is only RMB ¥50 (US $7.8) according to the current legislation. In addition, the managers in venues should take the opportunity to educate staff and enforce the mandate. An extensive and growing body of literature has shown that smoke-free policies have no economic impact on restaurants, pubs and other segments of the hospitality industry [18, 19]. Findings from the present study, along with the published findings [5, 6, 10, 15], indicate that a partial smoke-free legislation has had a weak impact on smoking cessation, but a comprehensive smoke-free legislation can substantially attenuate smoking prevalence without having negative economic impacts on the local businesses.

It was noteworthy that the implementation of a smoke-free legislation in Guangzhou did not lead to more smoking behaviors in homes. This finding is in agreement with the previous associations observed between smoke-free public places and a reduction in smoking practices at home [17, 20–22], and suggest that smoke-free public places did not lead to displacement of smoking from public places into homes. In addition, findings from the international tobacco control policy evaluation project in Europe and America also suggested that smoke-free public places facilitated rather than inhibited the introduction of smoke-free homes [20, 21]. These results supported the social diffusion hypothesis that more restrictive rules regarding smoking in public places would increase the likelihood that individuals would adopt voluntary home smoking restrictions [20]. The rate of smoking in homes in our study (from 91.0 % to 89.4 %) was much higher than those found in Albania (from 48 % to 33 %) and England (from 65 % to 55 %) [10, 17]. These findings further add support to the enactment of comprehensive smoke-free legislation in public places, and at the same time highlighted the urgent need for educational interventions and campaigns promoting smoking cessation at home and voluntary changes in home smoking rules, especially among those households with infants, children, and adult non-smokers.

Previous studies found that the implementation of the comprehensive smoke-free legislation in England did not have a substantial impact on smoking prevalence in adults [17, 23]. Despite the implementation of new tobacco control policies in Albanian, the smoking prevalence among males did not decrease, and smoking rates among females in general and in males aged 18–29 years continued to grow [10]. However, results from the 2002 to 2008 National Surveys from the US Census Bureau indicated that smoke-free laws and state tobacco control programs were effective strategies for curbing youth smoking [24]. Consistent with the above US study, the present study found that the reduction in the proportion of daily smokers was significant among 15–24 year olds for both genders, suggesting that a smoke-free legislation in Guangzhou is an effective strategy for curbing youth smoking. However, longer follow-up time may be needed to detect trends over time.

Previous research indicates that the implementation of comprehensive smoke-free legislation in Ireland and England had positive effects on quit attempts and quit successes respectively, and a partial smoke-free legislation in the Netherlands had no effect on quit attempts or quit successes [16, 25]. In Guangzhou, the quit ratios in most age groups increased after the implementation of a smoke-free legislation, but the quit ratios remained low compared to those in the countries with advanced tobacco control policies. The quit ratio was only 17.9 % in our evaluation survey, which was much lower than the 51.8 % found in the United States [26]. A study conducted in Hong Kong has suggested that smoke-free legislation that did not result in high rates of smoking cessation might displace smoking into homes [27]. Therefore, introduction of free tobacco cessation services, which are not currently available in Guangzhou, is urgently needed. In addition, quit rates might be further increased through better enforcement of the advertising bans and smoke-free legislation, as well as increasing the tax on cigarettes [10].

This study processes two strengths. The surveys were based on probability-based samples using standardized questions and allowed us to evaluate the different impacts of smoke-free legislation on smoking behaviors in full, partial and no smoking ban places at the same time. Additionally, to account for sample design and weighting in the estimation procedures, the statistical analyses were conducted with weighted data. However, some limitations should be considered when interpreting our results. First, the information was based on self-reports, and the findings may be susceptible to some bias. However, estimates obtained from population-based surveys that use self-reports are generally valid, apart from when there is a high demand for abstinence [28]. Second, the use of repeated cross-sectional data to assess the effectiveness of the smoke-free legislation may introduce bias, given that there may be differences in respondents between the two surveys. However, no significant differences in demographic characteristics (e.g., age and gender) were observed, and weighted data were used to adjust for the differences. Third, we found no significant differences between participants and non-participants in terms of sex, but the differences of age and smoking were uncertain since age and smoking were unable to be obtained from non-participants. This may have impacted the results for potential selection bias. Finally, this study lacks data to measure sufficiently what trends in smoking prevalence might have been in the absence of smoke-free legislation. Several national surveys conducted in China have indicated that in absence of a national smoke-free legislation, the smoking prevalence among adolescents had increased in the last decade in China [29–32]. Additionally, before the implementation of smoke-free legislation in Guangzhou, smoking prevalence among young women in this city increased from 1.2 % in 2008 [33] to 2.6 % in our baseline survey. This trend of escalation in smoking prevalence in Guangzhou and at a national level in China in the last decade suggests that the reduction in smoking prevalence observed in our 2011 sample is likely due to the implementation of smoke-free legislation in 2009.

## Conclusion

In conclusion, the partial smoke-free legislation implemented in Guangzhou, China has some effect on curbing smoking behaviors in places with a full smoking ban (such as cultural venues, public transport vehicles and government offices), reducing daily smoking prevalence in youth, and increasing the quit ratios in most age groups. However, smoking behaviors in public places and homes were still high and the quit ratios remained low after the implementation of a partial smoke-free legislation. These findings point out the urgent need for a comprehensive smoke-free legislation covering all public places in Guangzhou. Simultaneously, educational interventions and campaigns promoting smoking cessation and adoption of voluntary smoke free-home policies need to occur.
